# Social Environment of Older People during the First Year in Senior Housing and Its Association with Physical Performance

**DOI:** 10.3390/ijerph14090960

**Published:** 2017-08-25

**Authors:** Sinikka Lotvonen, Helvi Kyngäs, Pentti Koistinen, Risto Bloigu, Satu Elo

**Affiliations:** 1 Research Unit of Nursing Science and Health Management, Medical Research Center of Oulu, University of Oulu, P.O. Box 5000, 90014 Oulu, Finland; 2 Research Unit of Nursing Science and Health Management, Medical Research Center of Oulu, University of Oulu and Oulu University Hospital, P.O. Box 5000, 90014 Oulu, Finland; helvi.kyngas@oulu.fi (H.K.); satu.elo@oulu.fi (S.E.); 3 Faculty of Medicine, University of Oulu, P.O. Box 5000, 90014 Oulu, Finland; pentti.koistinen@outlook.com; 4 Medical Informatics and Statistics Research Group Oulu, University of Oulu, P.O. Box 5000, 90014 Oulu, Finland; risto.bloigu@oulu.fi

**Keywords:** social environment, older people, senior housing, relocation, physical performance, physical activity, walking speed, grip strength, chair stands

## Abstract

Increasing numbers of older people relocate into senior housing when their physical performance declines. The change in social environment is known to affect their wellbeing, providing both challenges and opportunities, but more information on the relations between social and physical parameters is required. Thus, we elicited perceptions of the social environment of 81 older people (aged 59–93 years, living in northern Finland) and changes in it 3 and 12 months after relocation to senior housing. We also measured their physical performance, then analysed associations between the social and physical variables. Participants reported that they had freedom to do whatever they liked and generally had enough contact with close people (which have recognized importance for older people’s wellbeing), but changes in their physical condition limited their social activity. Moreover, their usual walking speed, dominant hand’s grip strength and instrumental activities of daily living (IADL) significantly decreased. The pleasantness of the residential community, peer support, constraints on social activity imposed by changes in physical condition, meaningful activity at home and meeting close people all affected these physical performance parameters. Clearly, in addition to assessing physical performance and encouraging regular exercise, the complex interactions among social factors, physical performance and wellbeing should be considered when addressing individuals’ needs.

## 1. Introduction

When their physical performance declines, growing numbers of older people are relocating to senior housing, which provides customised environments for senior citizens with reduced functional capacities [1,2,3]. Such housing is managed by public or private elderly care organisations and offers services such as home and health care, social activities and physical activities. Most senior housing residents are women because on average they live longer than men [4,5]. The decision to relocate is influenced by a complex range of factors. It may be necessitated by requirements for services that support independent living, such as accessibility, a community environment and security [2,3,4]. However, relocation in later life may have stressful outcomes, such as a decline in physical performance [1,6], increases in instrumental activities of daily living (IADL) limitations and unsettling changes in social environment [7,8]. This is not surprising, as the environment is highly important for the wellbeing of older people [9,10], partly because the living environment often narrows in old age due to reductions in physical performance. Thus, the home environment and various aspects of surrounding locations strongly influence their wellbeing and quality of life [11,12]. The environment affects older people’s physical performance [13,14], individuals with low competence are most sensitive to environmental characteristics [10,14], and their wellbeing can be substantially improved by appropriate modifications of their environment to meet their needs [9,15]. The size of the home also affects the amount of daily physical activity; measured number of steps per day is directly related to the size of older people’s living space and is lower within retirement community residents than the community living older people [16].

There are numerous definitions of social environment. However, the definition used here is based on findings by Elo [9,17] that key elements of a supportive environment for older people that promotes their social wellbeing include a pleasant residential community, contact with family members, contact with supporting friends and availability of assistance. Important elements of a pleasant residential community include friendly people, social interaction and possibilities for meaningful activity. Contact with family members and friends enhances older people’s wellness and gives joy in their life. Relatives, friends and home care workers provide help and assistance. Friends give important peer support. Social relationships, getting support and a pleasant social environment all promote the wellbeing of older people [9,12,17].

Moreover, several studies have detected direct associations between older people’s social environment and both physical performance [13,14] and physical activity [4,10,15,18]. Lower extremity functionality (which reflects individuals’ ability to move, exercise and perform daily activities) is most important for older people’s physical performance [19,20] It incorporates cardiovascular endurance, muscular strength and exercise capacity [20,21]. Particularly senior housing residents have relatively lower extremity function [5]. Older people’s physical performance is inversely related to their sensitivity to environmental stressors, including social factors [4,13,14]. For example, lack of interest and company for outdoor mobility is reportedly associated with walking difficulties [14] and sedentary life in old age [22], while feeling familiar with the surroundings and living in a familiar social environment promote physical activity [4,14]. As older people in senior housing have mentioned walking as a priority exercise, the physical environment (non-slippery walkways, good outdoor areas, benches for resting, proximity to shops) is also an important factor [4]. Thus, when older people move to senior housing they may need support from close people or staff to get to know their new social environment, and it may be particularly important to organize social physical activities in the new environment for women, as they are more dependent than men on social networks [4,18]. If organisations do not support residents’ physical activity needs and preferences they tend to become inactive [23]. Support from family and staff as well as having friends who are physically active are both associated with the participation in physical activity [4,18]. Direct association has been shown between higher frequencies of talking to neighbours as well as more transportation and recreational walking among community dwelling older people [15]. Lack of sociability and low self-efficacy have shown to be barriers to physical activity among women in senior housing [4,5]. A recent study has noticed the importance of environment for the physical activity [4,5,10,14,24] and the physical performance [13,14] of older people. Up until now, to our knowledge, no studies have addressed the interactions between self-reported older people’s social environment and measured physical performance after relocation to senior housing.

Despite the findings outlined above, more empirical information about effects of transition to a new social environment in senior housing on older people’s physical performance is required. Thus, the aims of the study presented here were to elicit older people’s perceptions of their social environment and changes in it during the first year in senior-housing, and assess connections (if any) between those perceptions and changes in their physical performance.

## 2. Materials and Methods

### 2.1. Study Design and Data Collection

The empirical data were acquired in a longitudinal study, designed to elicit participants’ perceptions of their social environment and measure their physical performance 3 and 12 months after their relocation to senior housing. The participants were 81 older people, aged 59–93 years, who had all moved to one of 11 senior housing residences in northern Finland, owned by three private organisations.

In Finland senior houses are independent living facilities with rental apartments built specially for older people with reduced functional capacity [1,5,8,24]. Senior housing can be defined as an independent living community, where older people continue independent living in a sheltered environment with supportive services such as home care and health care as well as physical and social activities [1,24]. Senior houses do not offer 24/7 services, and residents are able to live in their own home with limited assistance [1]. The typical senior housing resident is a person 55–65 years or older who does not require assistance 24/7 but may benefit from the services, senior-friendly environment and increased social opportunities that senior houses offer [4,5]. The senior housing organisations are marketing their services and make their resident selections based on applications. The resident’s health status, functional capacity and their need for services affect the resident selection. The senior houses included in this study were barrier-free for physical activity (no thresholds, doors had automation, there were elevators) and were usually built near public services and recreational areas. Senior houses are mainly owned by national housing providers, associations or non-profit corporations and are medium size (50–100 apartments) [24]. The buildings do not differ technically from conventional apartments or row houses but are built to be accessible and barrier free and have common facilities for residents such as dining restaurant, rooms for physical and social activity, sauna and yard. Outside the senior houses were benches, but not in the neighbourhood recreational areas or along the streets.

Data were collected by face-to-face interviews during home visits 3 and 12 months after their relocation to these residences between June 2014 and December 2015. The inclusion criteria (apart from relocation to senior housing 3 months before the first interview and measurement round) were willingness to participate in the study, and capabilities to understand the aims and procedures of the study (did not have a memory disorder diagnosis), answer the multiple-choice questions reliably and engage in the physical capacity measurements. The target group consisted of 121 older people who met the first inclusion criterion (moving to senior housing 3 months before the first interview and measurement round), of whom 22 refused to participate in the study and 18 subjects did not meet other inclusion criteria. The sample of this study is typical of older people that have relocated in to senior housing in Finland, excluding residents with memory disorder.

### 2.2. Instruments

#### 2.2.1. Perceptions of the Social Environment

The modified instruments used to measure background variables, social environment and physical performance 3 and 12 months after the participants’ relocation to senior housing are presented in Table 1.

The Environmental Support instrument, used to assess the degree to which the participants’ environments promoted their wellbeing, is based on an instrument developed by Elo [17], which consists of 100 items and has been validated by expert evaluation, panel evaluation, principal component analysis, and confirmatory factor analysis [9,17]. In this study, we used a shortened version evaluated in 2014 by a panel of 21 experts, to ensure that no relevant concepts had been excluded. The shortened instrument consists of 30 items inviting Likert-type responses designed to evaluate the perceived supportiveness of the physical environment (9 items), symbolic environment (9 items), and social environment (12 items) for participants’ wellbeing. This study focuses on results pertaining to the social environment (Table 2 and Table 3), as measured in four sub dimensions: interpersonal relationships (4 items), getting support (3 items), pleasantness of the social environment (2 items), and feeling of social restrictiveness (3 items).

The items used were as follows: (1) I meet enough people close to me (family members, friends, neighbours); (2) I have enough contacts with people close to me (by phone or Skype); (3) I feel that people close to me (family, friends, neighbours) care about me; (4) People close to me bring joy into my life; (5) I get enough help from people close to me when I need it; (6) I get enough support from peers when I need it; (7) I have no problems when moving outside my home (going to the supermarket, health centre); (8) The community where I live (neighbours, people in the area where) is pleasant; (9) I have enough meaningful activities at home; (10) I feel that at home I have freedom to do whatever I like; (11) My life is too limited to the home environment; and (12) Changes in my physical condition have limited my social interaction. The response alternatives to the items were fully disagree, somewhat disagree, somewhat agree and totally agree.

#### 2.2.2. Background Variables and Physical Performance Parameters

Data on participants’ background variables, IADL performance, grip strength and lower body strength were collected using the Oldwellactive questionnaire designed to elicit elderly people’s perceptions of their wellbeing and wellness [25].

Their IADL limitations were measured using 11 items from a previously published IADL scale [26], asking “Do you cope independently with the following task?” (heavy housework such as washing the windows and house cleaning, ordinary housework, outdoor activities, shopping, finances, medicines, cooking, dressing, personal hygiene, bathing, using the toilet). Three responses were offered: yes (2 points); yes, but I have difficulties (1 point); and no, I need help from other people (0 point). Thus, overall scores ranged between 0 and 22, and were positively related to the participants’ self-rated independence in IADLs.

In addition, to assess the participants’ physical performance we assessed their grip strength, lower body strength and walking speed. Grip strength (of right and left hands) was measured to the nearest 1 kg using a Jamar handgrip dynamometer that participants were encouraged to squeeze as hard as possible.

Their lower body strength was measured by counting the number of full stands they could do in 30 s from a straight-backed chair [20]. In these tests, the participant initially sat in the middle of the chair (which was placed against a wall) with his or her back straight and feet on the floor. The participant was then asked to rise to a full stand and return to a fully seated position after a signal as many times and quickly as possible within 30 s. Participants were not allowed to use their hands or their thighs to push off the chair.

Participants’ walking speed was evaluated by timing a 4-m walk at their usual speed, one of three components of the Short Physical Performance Battery (SPPB) [19]. Each participant was asked to walk at his or her usual, comfortable speed across a 4-m course marked on the floor using the carpenters tape, with 60 cm buffer zones at both ends to allow participants to accelerate and decelerate. The time between each participant’s feet crossing the start and stop lines was timed with a stop watch, and the fastest of two timed walks was recorded. Each participant wore walking shoes, and used a walking aid if necessary. SPPB scores were not used in further analysis, except for the time in seconds to complete the course (which was assumed to be inversely proportional to the participant’s usual walking speed).

#### 2.2.3. Statistical Analysis

All analyses were performed using IBM SPSS Statistics 18 (IBM Corporation, New York, NY, USA). Baseline characteristics were described using frequencies, means and percentages. Likert scale was used to measure older people’s self-reported experience of social environment. The nonparametric Marginal Homogeneity significance test was used to examine differences in 3 and 12 months self-reported social environment. *p*-value 0.05 was chosen to indicate statistical significance. New variables were classified to show participant’s personal increase and decrease of self-reported social environment. The paired samples *t*-test was used to compare means in 3 and 12 months physical performance measurements. The One Sample *t*-test and nonparametric Wilcoxon test were used. New variables were calculated to show personal increase and decrease of physical performance measurements. The Independent Samples *t*-test was used to test association of self-reported social environment with change in measured physical performance.

#### 2.2.4. Ethical Issues

We received permission (29042014, 01092014, 02092014) to conduct the study from the three organisations that own the senior housing residences. We sent a letter to all the older people who had moved to those residences within the last 3 months, providing information about the study and inviting them to participate in it. After they had received the letter, they were called by phone and asked if they were willing to participate. Participants had the opportunity to ask for more information and discuss the study over the phone. All of the home visits and data collection procedures were performed by the same interviewer, who is a fully qualified physiotherapist. All participants were given written information about the study and signed a statement of informed consent during the first home visit. All the instruments used have been designed for assessing perceptions or measuring the performance of older people, and thoroughly validated.

## 3. Results

### 3.1. Background Characteristics of the Subjects

The background characteristics of the subjects are shown in Table 4. The subjects were 81 older people aged 59–93 years (*n* = 81) who had moved to senior-housing three months before the first interview and measuring round. Most participants were female (70%) with a mean age of 81 years. The most common medical conditions were coronary heart disease and musculoskeletal disease. Almost three quarters of participants (72%) used home care or personal care services.

### 3.2. Self-Reported Social Environment and Changes in It 3 and 12 Months after Relocation

#### 3.2.1. Pleasantness of Social Environment and Feeling of Social Restrictiveness

Frequencies of participants’ responses regarding the pleasantness of their social environment and feeling of social restrictiveness are shown in Table 2. In the second round of data collection, 12 months after their relocation, the strongest agreement (expressed by 87% of the participants) was for the statement that they had freedom to do what they liked in the senior housing. The feeling that the community is pleasant significantly increased (*p* = 0.010) during the first year in senior housing. Percentages agreeing that the community is pleasant rose from 32% after three months to 58% after 12 months. In addition, 37% of older people reported pleasantness of the community had increase, 15% reported that it had decreased and 53% reported no change. However, most participants (63% after 3 months and 70% after 12 months) agreed fully or somewhat that changes in their physical condition limited social interaction.

#### 3.2.2 Interpersonal Relationships and Getting Support

Percentages of participants’ responses regarding interpersonal relationships and getting support are shown in Table 3. After 12 months, the most frequently agreed feelings were that they had enough contact with close people by phone or Skype (87%) and that the close people cared about them (76%). The feeling that they met close people enough significantly increased (*p* = 0.023) during the first year in senior housing. Percentages agreeing that they met enough close people rose from 45% after three months to 53% after 12 months. In addition, 39% of older people reported that meetings with close people had increased, 18% reported it had decreased and 42% reported no change. Contact by phone or Skype with close people also reportedly increased significantly (*p* = 0.001); 3 and 12 months after the relocation 65% and 87% of the older people reported that they had enough phone or Skype contact with close people. However, numbers fully disagreeing that they could not easily move outside the home increased significantly (*p* = 0.019), from 22% after three months to 39% after 12 months. Moreover, 15% of the older people reported that problems moving outside had decreased, 35% reported they had increased and 49% reported no change, respectively, during their first year in senior housing.

### 3.3. Associations between Participants Perceptions of Their Social Environment and Physical Performance Parameters

Table 5 shows means of the measured physical performance parameters 3 and 12 months after relocation to senior-housing, and changes in them between these times. Walking speed (*p* = 0.002), dominant hand’s grip strength (*p* = 0.033), and IADL scores (*p* = 0.002) all significantly decreased. Mean grip strength of right and left hands decreased by 7.7% and 2.2%, respectively, mean walking speed by 22% and mean IADL scores by 6%. Numbers of chair-stands in 30 s also decreased slightly, but not significantly (*p* = 0.15).

**Figure 1:**
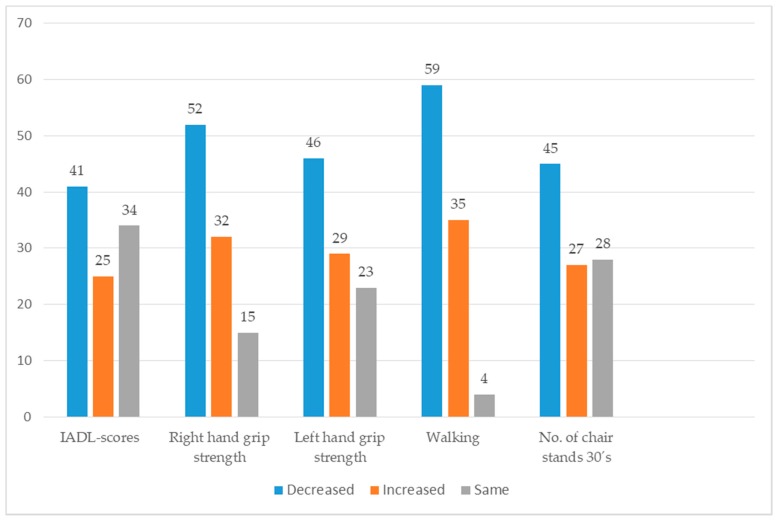
Frequencies (%) of participants whose physical performance parameters increased, decreased or remained the same during their first year in senior housing. IADL: instrumental activities of daily living.

Figure 1 shows percentages of participants whose measured physical performance parameters improved or declined during their first year in senior housing. The walking speed of 59%, 35% and 6% of participants decreased, increased and did not significantly change, respectively. Moreover, mean walking speed declined more (−5.033 s, sd 6.1, *n* = 11) among participants who reported that the pleasantness of their residential community had declined than among those reporting the residential community’s pleasantness had increased or remained the same (−0.574 s, sd 3.4, *n* = 60). An independent samples *t*-test indicated that this difference was significant: t(69) = 3.4, *p* = 0.004. Mean walking speed also declined more among participants who reported that peer support decreased or remained the same (−1.968 s, sd 4.3, *n* = 53) than among those reporting that peer support increased (0.805 s, sd 3.2, *n* = 18). An independent samples *t*-test indicated that this difference was significant: t(69) = −2.4, *p* = 0.015. In addition, mean walking speed declined more among participants who reported that physical limitations of social interaction decreased or remained the same (−1.76 s, sd 4.2, *n* = 45), than among those reporting reductions in physical limitations (−0.509 s, sd 0.4.1, *n* = 26). An independent samples *t*-test indicated that this difference was significant: t(69) = −2.2, *p* = 0.027.

The dominant hand’s grip strength of 52%, 32% and 16% of the participants decreased, increased and did not significantly change during the first year after relocation, respectively. It decreased more (2.823, sd 7.5, *n* = 47) among participants who reported that peer support increased or remained the same than among those reporting it decreased (−0.375, sd 4.34, *n* = 24). An independent samples *t*-test indicated that this difference was significant: t(67) = 2.3, *p* = 0.029. Mean dominant hand’s grip strength also decreased more (4.647, sd 6.6, *n* = 17) among participants who reported an increase in meaningful activity at home than among those reporting that amounts of such activities declined or remained the same (0.833, sd 6.5, *n* = 54). Independent samples *t*-tests indicated that this difference was significant: and t(69) = −2.1, *p* = 0.042.

IADL scores of 41%, 25% and 34% of the participants increased, decreased and remained the same, respectively. Mean IADL scores decreased more (1.536, sd 2.9, *n* = 28) among participants who reported increases in meetings with close people than among those reporting that amounts of such meetings declined or remained the same (0.372, sd 1.7, *n* = 43). An independent samples *t*-test indicated that this difference was significant t(41) = −2.0, *p* = 0.047).

## 4. Discussion

The Finnish population is ageing fast and the demand for senior houses will increase in the next twenty years [24]. Over the life course, a home that once was perfect as a living environment may turn out to be less optimal because of the decline of physical performance. Older people often relocate to senior housing when the functional decline occurs and they have environmental barriers at home that restrict their mobility so that they no longer are able to live independently [3].

In Finland senior housing means a living community for seniors who can live independently without the need of 24/7 assistance [24]. Senior houses are intended to be barrier-free, and should be suitable for moving around even with moving aids, such as walkers or wheelchairs [24]. Some senior houses are furnished with handrails, but most are designed so that railings are easy to install later [24]. In this study senior houses were privately owned rental apartments. Senior housing operators offer health care, meal and cleaning services as well as physical and social activities at extra fees. Senior houses have common facilities and services for residents such as a dining room, fitness room, sauna, staff and maintenance services [24].

This study explored social environment of older people and change in it 3 and 12 months after relocation to senior housing and it’s association to physical performance. The participants most strongly agreed, in their ratings of their social environment and social restrictiveness, that they had freedom to do what they liked. Thus, their social housing appears to uphold at least one core value of elderly care: the protection of rights to independence and privacy [23]. Moreover, associated feelings of freedom and privacy are normative elements of the elderly’s wellbeing, and strongly indicative of the absence of strong social pressure and (thus) possibilities to “be oneself” [9]. However, our participants also reported that changes in their physical condition limited social interaction. Similarly, 58% of older women surveyed in a previous study stated that health-related changes limited their social activities [1]. Limitations related to health and physical condition include reductions in mobility and restrictions of both activities and social interactions [2,15]. Feelings of limitation can be reduced by help from home care staff and close people. Various kinds of gerontechnology (moving aids, various support rails, less slippery floors, non-slip shoes, automatic lights, safety watch with emergency button) can also help older people to overcome limitations imposed by reductions in their physical performance [9]. Moreover, there are reported correlations between social diversity and older people’s walking capacities [15]. Thus, these findings indicate that more efforts should be made to encourage residents of senior housing (at least the housing considered here), especially the frail ones, to be active and engage in both social and physical activities [23]. Introduction of more appropriate gerontechnology and facilities for suitable activities may also be important (possibly more important than active encouragement by nursing staff to be active, which could potentially compromise feelings of freedom, privacy and lack of social pressure).

Participants’ feeling that the community was pleasant significantly increased during their first year in senior housing. According to previous studies, socialization requires the greatest adaptive efforts after moving to senior housing [8], and neighbourhood social capital is beneficial for the wellbeing of elderly people, especially women [12]. Key features of a pleasant community include friendly people, good interactions with neighbours, family and friends, and group activities, all of which are associated with a positive mood [9,17]. Thus, the residences of our participants appear to meet these major needs of older people, despite the scope noted above for increasing encouragement of activities which would also boost social interactions and hence, probably, a positive sense of community.

Our findings regarding contact with close people are intriguing in several ways. Older people feel cared for when family members visit and keep in touch with them regularly, even keeping in touch by phone can be enough. This is particularly important for women, and feelings that close people do not care is associated with loneliness [9,17]. Our participants generally felt they had enough contact with close people by phone or Skype, and that close people cared about them. Thus, the residences clearly did not hinder such contact. In addition, feelings that they had enough meetings with close people, and enough contact with them by phone or Skype, increased significantly during their first year. Families and close people are often involved in the relocation of older people, to help them integrate in the new environment and choose the services they need [8]. This could explain feelings of sufficient contact in early stages, but it does not readily explain why the feelings increased during our participants’ first year after relocation. Moreover, another study found no significant difference in numbers of older women’s visits by families and friends before and four months after relocation to senior housing [1]. Motivation and support from family members are clearly important in changes of life situations and integration in senior housing [4,9]. However, our results indicate that other social factors (notably a pleasant community and good social interactions with other people, as delineated above) may reduce the need for visits by close people, and hence increase the feeling that one has “enough” contact with family and friends.

Our participants’ movements outside their new home, for example going to the supermarket or health centre independently, significantly decreased during their first year in senior housing, and their lower extremity functionality was initially poor and subsequently declined. Clearly, these are concerns as older people’s mobility is linked to their ability to cope with various aspects of the neighbourhood environment. Moreover, familiarity with surroundings decreases risks of walking limitations and fear of falling, while feelings of insecurity increase them [14]. The walking speed (which is a key factor in maintaining physical independence outdoors as well as associated with lower extremity muscle strength) of participants decreased significantly. In this study the measured lower extremity performance parameters of the participants were poorer than average for the home-living population of the same age [20,27,28]. Unfamiliar surroundings in a new environment are reported to increase walking limitations [4,14,18]especially among older people with poor lower extremity performance [14]. Knowing the neighbourhood was an important indicator of a successful transition to senior housing for women [4]. The outdoor environment is an important factor because walking is a priority exercise of older people [4]. Family, friends and staff should support older people’s physical activity in the new surroundings [4,18].

Longing for the old neighbourhood and old neighbours may also reduce older people’s physical activity outside the home in new social environments [4]. Clearly, it is not possible to relocate old neighbourhoods and neighbours. However, these problems can potentially be addressed by actions such as providing benches to rest on in the neighbourhood of senior housing [4], introducing appropriate gerontechnology (walking aids and mobile phones) and striving to ensure that the social environment is as pleasant and supportive as possible. In this study senior houses were built near public services and recreational areas. There were benches in the yards of senior houses where residents could engage in social interactions, but there was a lack of benches for resting on walking trips in the neighbourhood. Increasing the safety of the new neighbourhood environment with benches and arranging company on walking trips would be needed [14].

In accordance with expectations, measured physical performance parameters of the participants were poorer than average for the home-living population of the same age (data not shown, but for details compare data presented here with corresponding values in [20,27,28] Moreover, their measured walking speed, IADL performance and dominant hand’s grip strength significantly decreased during the first year in senior housing, and numbers of chair stands they could do in 30 s decreased slightly. Similarly, a previous study found that older people living in senior housing in Georgia, USA were less physically active than community-dwellers of the same age. For example, they took 3000 fewer steps per day (and this variable was related to their living space and functional capacity) [16]. Moreover, relatively physically active older people in senior housing reportedly have fewer functional limitations at 12 months follow-up than those who are less active [5]. Nevertheless, functional limitations inevitably increase with old age and result in a decline of physical performance, even after relocation to senior housing with fewer environmental barriers [3]. Perhaps partly for this reason another study found that very old people (71–100 years old) living in Swedish residential care facilities spent most of their time in their apartment where they slept, dozed or independently engaged in self-care [23], and light housework is reportedly a primary type of physical activity in retirement communities [5]. These findings indicate a need to consider several factors. Senior housing should clearly provide an environment tailored to the needs of older people, whose physical performance will gradually decline. Smaller dwellings and closeness of services may make living easier for the older people, but it may also reduce their daily living activities, amounts of physical activity and physical performance [16]. Thus, a good balance should be sought between convenience and opportunities for activities, although establishing such balance is far from straightforward as the optimum point gradually changes as people age.

It is also clearly important to consider social factors, as (for example) the walking speed of participants who reported reductions in pleasantness of the community, peer support or physical limitations of social interactions declined significantly more than that of participants who reported increases in these social variables during their first year in senior housing. Accordingly, lack of company has been shown to be a significant barrier for older people’s physical activity, while supportive and motivating company promotes it [22], especially in new surroundings. Peer support also enables people to share health and mobility problems (and ways to address them) with people in similar situations, while lack of peer support and friends may decrease physical activity outside the home [9,15]. Moving to senior housing poses both challenges and opportunities, offering scope to forge new relationships while maintaining previous external social contacts [8], but residents who (for example) spend most of the time in their apartments will miss opportunities to participate in group activities and social interactions with others. Thus, it is important to consider the needs of older people with varying levels of frailty and physical capacity, not only those who can readily participate in group activities and move outside their home independently [23].

Unsurprisingly, mean grip strength of our participants’ dominant hands decreased. Reasons for this may include declines in health [22], living space [16] and daily activities [5] after relocation, in addition to gradual decline with increasing age. Moreover, in contrast to the findings regarding walking speed, grip strength of the dominant hand decreased significantly more among older people whose self-reported peer support increased or remained stable than among those reporting reductions in peer support. This may seem counter-intuitive. However, poor grip strength is a strong predictor of dependency in daily living activities, cognitive decline [29] and more general impairment of physical performance [30]. In addition, the correlation with peer support may be simply because it is easier to establish such support among people with the same health problems, religion or life situations, such as older people in senior housing with declining grip strength [2,8,17]. Thus, to optimise the social environment, senior housing facilities should promote peer-exercise networks and physical exercise groups to support residents’ physical performance [5].

IADL performance decreased significantly more among participants indicating that meetings with close people increased than among those who reported that frequencies of such meetings decreased or remained the same. However, this could be partly because people with strong declines in IADL performance had the greatest needs of visits by close people. Older people’s IADL performance is associated with biological ageing, chronic diseases, physical and cognitive performance, living environment and gender, as IADL difficulties increase with age more among women than men [31]. Relocation causes short-term stress and IADL performance declines more among older people who move because of health reasons than among those who move for other reasons. For older people who move for health reasons, adaptation to senior housing should be facilitated by assistance of caregivers and formal support services designed to help them function in their new environment [7]. However, as mentioned above, senior housing environments may hasten the dependence process through reductions in living space and provision of supportive services (household help and personal care) [5], so efforts should be made to balance such changes with promotion of performance-enhancing activities.

### Strengths and Limitations

A strength of the study is that all the interviews and physical performance measurements were conducted by the same person, who was a physiotherapist and had experience of the instruments used and home visits. In addition, we used both self-reported and objectively measured information to evaluate older people´s social environment and its association with physical performance during their first year in senior housing. This support the validity of the study. Use of face-to-face interviews is a further strength, as they are regarded as more suitable for older people than questionnaires, and provide possibilities for interviewees to discuss potential ambiguities, misunderstandings or other issues with the interviewer [32]. The Environmental Support instrument also has strengths, in providing a means to access older people’s perceptions via an interviewee-centred approach. A limitation is that our results are based on quite a small sample of elderly Finnish people, mostly women, living alone. However, participants in other studies of older people who had relocated to senior housing were mostly women living alone [4,5], which increases the comparability of our findings with those of previous studies. People with cognitive impairments were excluded from our sample of older people (all volunteers), which limits generalizability, but presumably improves the reliability of the self-reported perceptions. It is also possible, that the frailest older people in the focal residences did not participate in the study, because of their health impairments. In addition, the restriction to people living in northern Finland clearly limits the generalizability of the findings, and it should be noted that the data were collected in different seasons, so seasonal variations could have influenced the participants’ physical performance and self-reported perceptions of their social environment.

## 5. Conclusions

The presented exploration of the social environment of Finnish older people during their first year in senior housing, and its associations with physical performance parameters, showed that they generally felt they had freedom to do whatever they liked and enough contact with close people. However, they reported limitations in moving outside the home, and there were significant reductions in their dominant hand’s grip strength, walking speed and IADL performance. Furthermore, reductions in pleasantness of the residential community and peer support, as well as limitations of social activity due to changes in physical condition, were associated with reductions in walking speed. In addition, increases in peer support and meaningful activities at home were associated with reductions in dominant hand’s grip strength, and increases in meeting close people were associated with reductions in IADL performance.

Older people relocate to senior housing when their physical performance is declining, and the new social environment presents both challenge and opportunities, but residents who (for example) spend most of the time in their apartments will miss opportunities to participate in group activities and social interactions with others. Thus, it is important to consider the needs of older people with varying levels of frailty and physical capacity, not only those who can readily participate in group activities and move outside their home independently. Our findings indicate that more efforts should be made to encourage residents of senior housing, especially frail residents, to be active and engage in both social and physical activities. Thus, regular assessment of physical capacities, together with regular exercise designed to maintain muscle strength and balance are important to minimise decline of physical performance. Family members, close people and staff all play important roles in helping adaptation to the new environment and motivating older people to be physically active in new surroundings. Training for staff to promote residents’ physical activity is also required. However, introduction of more appropriate gerotechnology and facilities for suitable activities may be even more important, partly because excessive encouragement by nursing staff could potentially compromise feelings of freedom and privacy.

Finally, senior housing should clearly provide an environment tailored to the needs of older people, whose physical performance will gradually decline. Smaller dwellings and closeness of services may make living easier for the older people, but it may also reduce their daily living activities, physical activity and physical performance. Thus, a good balance should be sought between convenience and opportunities for activities, although establishing such balance is far from straightforward as the optimum point gradually changes as people age.

## Figures and Tables

**Table 1:** The modified instruments used to measure participants background characteristics, perception for their social environment and physical performance 3 and 12 months after relocation to senior housing.

Instruments	Assessment Categories and Items				
Environment wellness instrument (self-reported social environment)	Pleasantness of social environment (2 items)	Feeling of social restrictiveness (3 items)	Interpersonal relationships (4 items)	Getting support (3 items)	
Oldwellactive questionnaire and SPPB (background characteristics and physical performance)	Background characteristics (10 items)	Do you cope independently with the following IADL tasks? (11 items)	Grip strength, Kg (Jamar dynamometer)	Lower body strength, No. of chair stands in 30 s	Usual walking speed, 4 m (SPPB)

SPPB: Short Physical Performance Battery; IADL: Instrumental activities of daily living.

**Table 2:** Self-reported pleasantness of social environment and feelings of social restrictiveness and changes in these variables 3 and 12 months after relocation to senior housing.

Pleasantness of Social Environment and Feelings of Social Restrictiviness	3 Months		12 Months		Increased	Decreased	No Change	Significance *
	*n*	(%)	*n*	(%)	*n* (%)	*n* (%)	*n* (%)	*p*-Value *
Pleasantness of social environment								
*“The community where I live is pleasant”*								
Fully agree	23	(32)	41	(58)				
Somewhat agree	44	(62)	25	(35)				
Somewhat disagree	4	(6)	5	(7)				
Fully disagree	0	(0)	0	(0)				
					26 (37)	11 (15)	37 (53)	0.010
*“I have enough meaningful activities at home”*								
Fully agree	29	(41)	29	(41)				
Somewhat agree	17	(24)	21	(30)				
Somewhat disagree	20	(28)	12	(17)				
Totally disagree	5	(7)	9	(12)				
					17 (24)	18 (25)	36 (51)	1.000
**Feeling of social restrictiveness**								
*“I feel that at home I have freedom to do whatever I like”*								
Fully agree	58	(82)	62	(87)				
Somewhat agree	9	(13)	2	(3)				
Somewhat disagree	1	(1)	5	(7)				
Totally disagree	3	(4)	2	(3)				
					10 (14)	8 (11)	53 (77)	0.803
*“My life is too limited to the home environment”*								
Fully agree	24	(34)	21	(30)				
Somewhat agree	21	(30)	28	(39)				
Somewhat disagree	13	(18)	15	(21)				
Fully disagree	13	(18)	7	(10)				
					20 (28)	23 (32)	28 (39)	0.499
*“Changes in my physical condition have limited my social interaction”*								
Fully agree	27	(38)	27	(38)				
Somewhat agree	18	(25)	23	(32)				
Somewhat disagree	5	(7)	4	(6)				
Fully disagree	21	(30)	17	(24)	12(17)	17 (24)	42(59)	0.035

* According to the Marginal Homogeneity test.

**Table 3:** Self-reported interpersonal relationships and getting support and changes in these variables 3 and 12 months after relocation to senior housing.

Interpersonal Relationships and Getting Support	3 Months		12 Months		Increased	Decreased	No Change	Significance *
	*n*	(%)	*n*	(%)	*n* (%)	*n* (%)	*n* (%)	*p*-Value *
Interpersonal relationships								
*´´I meet enough people close to me“*								
Fully agree	32	(45)	38	(53)				
Somewhat agree	12	(17)	21	(30)				
Somewhat disagree	23	(32)	9	(13)				
Fully disagree	4	(6)	3	(4)				
					28 (39)	13 (18)	30 (42)	0.023
*“I have enough contact with close people (phone, Skype)”*								
Fully agree	46	(65)	62	(87)				
Somewhat agree	12	(17)	4	(6)				
Somewhat disagree	11	(16)	5	(7)				
Fully disagree	2	(3)	0	(0)				
					21 (30)	5 (7)	45 (63)	0.001
*“I feel that people close to me care about me”*								
Fully agree	54	(76)	54	(76)				
Somewhat agree	14	(20)	13	(18)				
Somewhat disagree	2	(3)	1	(1)				
Fully disagree	1	(1)	3	(4)				
					7 (10)	7 (10)	57 (80)	0.631
*“People close to me bring joy into my life”*								
Fully agree	48	(68)	45	(63)				
Somewhat agree	20	(28)	24	(34)				
Somewhat disagree	3	(4)	2	(3)				
Fully disagree	0	(0)	0	(0)				
					10 (14)	14 (20)	47 (66)	0.715
**Getting support**								
*“I get enough help from people close to me when I need it”*								
Fully agree	51	(72)	46	(65)				
Somewhat agree	10	(14)	16	(23)				
Somewhat disagree	9	(13)	7	(10)				
Fully disagree	1	(1)	2	(3)				
					13 (18)	16 (23)	42 (59)	0.529
*“I get enough support from peers, when I need it”*								
Fully agree	19	(27)	19	(27)				
Somewhat agree	16	(23)	17	(24)				
Somewhat disagree	20	(28)	18	(25)				
Fully disagree	16	(23)	17	(24)				
					18 (25)	24 (34)	39 (55)	1.000
*“I have no problems when moving outside home”*								
Fully agree	26	(37)	20	(28)				
Somewhat agree	11	(16)	9	(13)				
Somewhat disagree	18	(25)	14	(20)				
Fully disagree	16	(22)	28	(39)				
					11 (15)	25 (35)	35 (49)	0.019

* According to the Marginal Homogeneity test.

**Table 4:** Background characteristics of older people three months after relocation to senior housing (*n* = 81).

Background Characteristics	*n*	%
**Age (years)**		
55–64	4	5
65–74	9	11
75–84	42	52
85–94	26	32
**Gender**		
Female	57	70
Male	24	30
**Marital status**		
Married	27	33
Unmarried	7	9
Widowed	30	37
Divorced	17	21
**Housing**		
Lived alone	61	75
Lived with someone (cohabited)	20	25
**Children**		
Has children	68	84
No children	13	16
**Service use**		
No services	23	28
Services	58	72
**Financial situation**		
Very good	2	3
Good	23	28
Moderate	53	65
Poor or very poor	3	4
**Mood at the moment**		
Very good	1	1
Good	22	31
Average	44	62
Bad	4	6
**Worried about being depressed or hopeless in the last month**		
Yes	24	34
No	47	66
**Diseases**		
Coronary heart disease	72	88
Musculoskeletal disease	59	73
Neurological disease	21	26
Diabetes	14	17
Respiratory organ disease	13	16
Cancer	4	5

**Table 5:** Measured physical performance parameters and changes in them 3 and 12 months after moving to senior housing.

Measured Physical Performance	3 Months	12 Months	3–12 Months	Significance *
	Mean	Mean	Change	*p*-Value
Usual walking 4 m (s) (Walking speed m/s)	6.27	7.64	+1.37	0.002
	0.63	0.52	–0.11	
Grip strength, right hand (kg)	22.94	21.20	–1.74	0.033
Grip strength, left hand (kg)	20.80	20.28	–0.52	0.410
IADL scores	14.65	13.82	–0.83	0.002
No. of Chair stands in 30 s’	6.58	6.13	–0.45	0.150

* According to the Paired Samples *T*-test. IADL: instrumental activities of daily living.
